# Molecular Genotyping (SSR) and Agronomic Phenotyping for Utilization of Durum Wheat (*Triticum durum* Desf.) Ex Situ Collection from Southern Italy: A Combined Approach Including Pedigreed Varieties

**DOI:** 10.3390/genes9100465

**Published:** 2018-09-20

**Authors:** Stefania Marzario, Giuseppina Logozzo, Jacques L. David, Pierluigi Spagnoletti Zeuli, Tania Gioia

**Affiliations:** 1 Scuola di Scienze Agrarie, Forestali, Alimentari ed Ambientali, Università degli Studi della Basilicata, viale dell’Ateneo Lucano 10, 85100 Potenza, Italy; stefy.85s@libero.it (S.M.); pierluigi.spagnoletti@unibas.it (P.S.Z.); tania.gioia@unibas.it (T.G.); 2 Montpellier SupAgro, UMR AGAP Amélioration Génétique et Adaptation des Plantes méditerranéennes et Tropicales, 34060 Montpellier, France; jacques.david@supagro.fr

**Keywords:** *Triticum durum* (Desf.), germplasm, simple sequence repeats, morphophysiological traits, genetic diversity

## Abstract

In South Italy durum wheat (*Triticum durum* Desf.) has a long-time tradition of growing and breeding. Accessions collected and now preserved ex situ are a valuable genetic resource, but their effective use in agriculture and breeding programs remains very low. In this study, a small number (44) of simple sequence repeats (SSR) molecular markers were used to detect pattern of diversity for 136 accessions collected in South Italy over time, to identify the genepool of origin, and establish similarities with 28 Italian varieties with known pedigree grown in Italy over the same time-period. Phenotyping was conducted for 12 morphophysiological characters of agronomic interest. Based on discriminant analysis of principal components (DAPC) and STRUCTURE analysis six groups were identified, the assignment of varieties reflected the genetic basis and breeding strategies involved in their development. Some “old” varieties grown today are the result of evolution through natural hybridization and conservative pure line selection. A small number of molecular markers and little phenotyping coupled with powerful statistical analysis and comparison to pedigreed varieties can provide enough information on the genetic structure of durum wheat germplasm for a quick screening of the germplasm collection able to identify accessions for breeding or introduction in low input agriculture.

## 1. Introduction

As part of a worldwide effort large collections of plant genetic resources have been established to meet future needs for agriculture and breeding and maintained ex situ in genebanks [1] but, due to the lack of genetic information, their effective use has been fairly small [2].

Durum wheat (*Triticum turgidum* L. ssp. *durum* Desf., 2*n* = 4x = 28; AABB genomes) is one of the most widely cultivated cereal species worldwide. It represents a major renewable resource for food, feed, and industrial raw materials [3] and is traditionally grown around the Mediterranean, mainly in Italy, Spain, France, Greece, West Asian, and North African countries [4]. The recent history of durum wheat has been characterized by the replacement of landraces and the improvement of modern cultivars by inbred varieties in which dwarfing genes were introduced (second part of the 20th century) and adapted to favorable lands where high input agriculture is practiced [5,6]. So, the accessions collected might include germplasm from different genepools: cultivated varieties (cultivars) in current use; newly developed varieties; obsolete cultivars, primitive cultivars (landraces); but also, special genetic stocks (including elite and current breeders’ line and mutants).

Studies on genetic diversity within ex situ germplasm collections are needed since their effective utilization for breeding relies on the ability to identify accessions with a given characteristic or possibly with the desired gene. The passport data, when available, are useful information since they relate the accession to where and when it was obtained/collected, thus providing hints on the more likely genetic characteristics or sometimes the gene pool of origin. Phenotypic characterization data of agronomic interest both for qualitative and quantitative traits (e.g., spike shape, spike density, seed color, days to heading, plant height, and spike length) are very useful but for the characterization of large collections a huge amount of money and human resources are needed and must deal with little polymorphism, low heritability, and genotype by environment interaction [1]. In addition, morphological traits selected for scoring might end up assessing diversity for a small, not representative amount of the whole genome.

The advances in molecular genotyping provide tools easier to handle the study variation in germplasm collections [7]. DNA molecular markers have been used to assess the amount of variation and describe the genetic relationships among accessions [8]. For their high polymorphism, codominance and locus specificity, simple sequence repeats or microsatellite (SSRs) markers have proved to be highly efficient molecular tools for the characterization of durum wheat germplasm collections [9,10,11,12,13,14].

To date most studies on Italian durum germplasm have analyzed collections including old and new elite varieties for morphophysiological and qualitative traits [15,16], and the use of molecular markers was focused on temporal trends of diversity [8,17,18], relatedness among genotypes [10], genetic structure [19], also in comparison to *Triticum turgidum* L. subspecies [13].

In a recent study [18] a panel of 370 durum wheat genotype including 35 Italian genotypes (29 elite varieties and six landraces) where genotyped using 500 simple sequence repeats (SNPs) markers. All varieties were obtained from crosses and not by pure line selection, and, except for one (Capeiti 8), were all “modern” released after 1974 when Creso, the first Italian successful dwarf variety, was released. 

In Italy durum wheat has a long-time tradition of growing and breeding and accessions collected in Southern Italy, now preserved ex situ, are a valuable genetic resource. A large number of Italian and some north African landraces were grown in Italy since the end 1800 and after 1920 [20] were gradually substituted by the pure line varieties selected from landraces from Italy, but also from Syria-Palestinia and north Africa (Lybia) [21]. Almost all the genetic material was from *Mediterraneum typicum* and *Syriacum typicum* groups [22,23] where Syriacum with erect leaves and short head, did show a shorter plant, earlier heading, higher tillering, than Mediterraneum. 

New varieties were then obtained from crosses between lines from Mediterraneum (Senatore Cappelli) and Syriacum (Aziziah, Eiti, Sinai, and Tripolino) groups (1950–1965), from the introgression from tetraploid and hexaploid species and mutagenesis (1965–1973), the introduction of Norin10 dwarfing genes (1970–1980) and of new germplasm mostly from CIMMYT (Centro Internacional de Mejoramiento de maiz y trigo, Messico) and from recombination of the whole available tetraploid gene pool (1980–1990), followed [24,25,26,27,28]. 

Luckily, as the result of extensive germplasm collecting expedition programs that did start in 1948, durum wheat germplasm from inland South Italy was collected; also in the most remote areas and preserved ex situ collections [21]. Today these landraces/old varieties, along with their breeding potential, are of interest for cultivation in marginal areas and in organic or low-input agricultural systems since the typical local products pay a premium price and ex situ collection can provide this germplasm. The detailed passport data that are available (e.g., accession name, collection site, geographical origin, and collecting year) only in few cases include a “variety” name. Almost no information is available to identify the gene pool of origin and length of time for evolution at the collection site or about their genetic characteristics, which are important because of the relevant role of durum wheat breeding in Italy. 

In this study a strategy combining molecular genotyping, phenotyping, and the use of pedigreed varieties is proposed to characterize a durum wheat ex situ germplasm collection: (i) a small number of SSR molecular markers were used to detect pattern of diversity for 136 accessions collected in South Italy over time; (ii) to identify the genepool of origin of these ex situ accessions the same molecular markers were used to establish similarities with 28 Italian varieties with known pedigree; and (iii) phenotyping was than conducted for 12 morphophysiological characters of agronomic interest and the structure of diversity compared to identify the most diverse accessions for breeding purposes or for use in low input agriculture. 

The results show that a small number of molecular markers and little phenotyping coupled with powerful statistical analysis and comparison to pedigreed varieties can provide enough information on the genetic structure of durum wheat germplasm for a quick screening of the ex situ germplasm collection, in order to identify accessions for breeding or introduction in low input agriculture.

## 2. Methods

### 2.1. Plant Material 

The analyzed durum wheat germplasm included 136 accessions collected in Southern Italy from 1947 to 2003, now preserved ex situ in genebanks, and of 28 elite varieties cultivated in the past decades in Italy and representative of the Italian breeding programs. 

Seed samples for all accessions were obtained by the Institute of Plant Genetics and Crop Plant Research (IPK), Germany; Centro per la Salvaguardia delle Risorse Genetiche Vegetali “Pierino Iannelli”, Università degli Studi della Basilicata (Unibas), Italy; and the Istituto di Bioscienze e Biorisorse del Consiglio Nazionale delle Ricerche (IBBR-CNR), Italy. Passport information (e.g., accession name, collsite, geographical origin, and collecting year) was available for all the accessions. In only a few cases the accession was also identified with a “variety” name. Luckily the accessions of eight very old “varieties” have been collected in 1950 from an institution well-known for durum wheat breeding (Stazione Sperimentale di Granicoltura, Catania). But in most cases, none of the information available was useful to distinguish “landraces” from varieties or to identify the gene pool of origin and length of time for evolution at the collection site. The seeds of 28 varieties were obtained from the Centro di Ricerca per la Cerealicoltura-Foggia (CREA-CER), Italy. Year of release, registered pedigree, and breeder for each elite variety were recorded. A complete list of the accessions analyzed is available in Supplementary Materials file 1: Tables S1 and S2. 

For the purpose of the study, accessions were subdivided into groups according to the year in which the collecting expeditions took place: Group1 (number of accessions = 37) comprised of accessions collected in the period 1947–1950; Group2 (number of accessions = 77) included accessions collected from 1973 to 1982; and Group3 (number of accessions = 22) consisted of accessions collected during the period 1983 to 2003. 

According to the 28 varieties’ release year, two temporal groups were identified: (i) “old and intermediate” group (1915–1975) including “old” genotypes selected from indigenous and exotic landraces, and “intermediate” genotypes selected from crosses or mutagenesis involving old materials (*n* = 9); (ii) “modern” including genotypes released after the 1980s, mostly selected from crosses between CYMMIT (International Maize and Wheat Improvement Center) breeding lines and materials belonging to the former set of varieties (*n* = 19). 

### 2.2. Molecular Characterization

In order to estimate molecular diversity, the germplasm collection was characterized using 44 SSRs markers. All the nuclear SSRs markers were selected on the basis of their known genetic locations to give a uniform coverage for all seven linkage groups as well as their temperature of annealing (T_an_) and degree of polymorphism [29,30,31,32]. More information about the SSR markers used, including the designation, chromosome location, primer pair sequences, the repeat motif, and the allele size range of the amplified loci are available in Supplementary Materials file 1: Table S3.

### 2.3. Genomic DNA Extraction and Genotyping 

Genomic DNA was extracted from leaf tissues at the tillering stage of greenhouse-grown plants using the CTAB (Cetyltrimethylammonium bromide) method [33] with minor modifications. The purity and concentration of DNA was determined spectrophotometrically at 260 and 280 nm by using Nano Drop^®^ Spectrophotometer ND 1000 (NanoDrop Technologies, Wilmington, DE, USA). The DNA samples were diluted to a final concentration of 20 ng/μL with TE (Tris-EDTA) for the following SSRs analysis. 

Multiplex PCR (two to five SSRs primer pairs with the same annealing temperature in one PCR reaction) and multiple sample loading (loading more than one PCR reaction in each well) were used. The reverse primer for each locus was labeled with one of the three fluorophores (6FAM, NED, or HEX) and PCR amplification was performed with a final volume of 20 µL containing 30 ng of genomic DNA, 0.2 mM of each dNTP, 2 nM MgCl_2_, 1–4 pM of each primer, and 0.5 U of Taq DNA polymerase. PCR was carried out as follows: after 5 min at 94 °C, 35 cycles were performed with 30 s at 94 °C, 30 s at either 55 or 60 °C (depending on the locus), and 1 min at 72 °C, followed by a final extension step of 30 min at 72 °C. The PCR products were detected by capillary electrophoresis using an ABI PRISM 3130xl Genetic Analyzer (Applied Biosystems, Foster City, CA, USA) and analyzed using the GENEMAPPER V3.7 genotyping software (Applied Biosystems).

### 2.4. Morphological Characterization

Three plants for each of the 136 accessions from ex situ genebanks and 28 varieties were grown in the greenhouse at the Università degli Studi della Basilicata (Potenza, Italy) during the growing season of 2012 to 2013. Heading date (expressed as days after 10 April) was recorded when the culms showed emerging spikes and plant height (excluding spike) was measured at harvest time. Individual plants were harvested manually in July 2013 and ten quantitative morphological traits were scored according to descriptors for wheat defined by IBPGR (International Board for Plant Genetic Resources) [34]: spike length (cm), spikelets number/spike (n), fertile spikelets number/spike (n), kernel length (mm), kernel width (mm), kernels number/spike (n), kernels weight/spike (g), 1000 kernels weight (g), kernels number/spikelet (n), and kernels number/fertile spikelets (n). Three characters were calculated: (i) thousand kernels weight (g) as (kernels weight per spike/kernel number per spike) × 1000; (ii) kernels number per spikelet as kernel number per spike/spikelet number per spike; (iii) kernels number per fertile spikelet as kernel number per spike/fertile spikelet number per spike. 

### 2.5. Statistical Analysis

Summary statistics for each SSRs locus, including the number of alleles detected and the gene diversity or unbiased expected heterozygosity (H_e_ [35]) were calculated using the software Genetix V4.05 [36].

The level of variation in different germplasm types (landraces and elite varieties) and within temporal groups of accessions was evaluated in terms of number of alleles per locus (N_a_), gene diversity, or unbiased expected heterozygosity (H_e_ [34]) and allelic richness (R_s_ [37]). All these indices were calculated using FSTAT 2.9.3.2 software [38]. For computing R_s_, FSTAT implements the rarefaction method to trim the unequal accessions number to the same standardized sample size, a number equal to the smallest sample across the population. Overall differences in the expected heterozygosity and allelic richness between temporal groups of accessions were assessed for significance using FPTestR software [39]. Population structure within the germplasm collection was examined by first applying the Discriminant analysis of principal components (DAPC) [40], a new multivariate method designed to identify and describe clusters of genetically related individuals. This approach allows extracting rich information from genetic data, providing assignment of individuals to groups, a visual assessment of between-population differentiation, and contribution of individual alleles to population structuring. Discriminant analysis of principal components does not assume unrelatedness, and therefore, potentially closely related individuals can be included in the analysis. The method relies on allele data transformation using principal component analysis (PCA) as a prior step to discriminant analysis (DA). Discriminant analysis of principal components was performed using the adegenet package [41] in R (R development Core Team, 2009). The optimal number of clusters was determined using the *find.clusters* function which implements successive K-means clustering. The rate of decrease of the bayesian information criterion (BIC) was visually examined, and the number of clusters was determined as the value of K above which BIC values decreased or increased only subtly. We then applied the *dapc* function to describe the relationship between the inferred groups. This function constructs synthetic variables and discriminant functions (DFs) that maximize variation between while minimizing variation within groups and computes coordinates along these functions for each individual. In order to obtain reliable group membership probabilities and to avoid overfitting, we retained only the four first principal components (PCs) from the preliminary data transformation step (indicated to be the optimal number based on the *optim.a.score* function).

The assignment of each accession to the different clusters was examined taking into account the germplasm type (accessions or varieties) and the temporal group to which each genotype belonged.

The model-based approach implemented in the software package STRUCTURE [42] was then further applied to infer population structure. Initially, the number of subgroups (K) was set from two to ten. Twenty independent simulations were performed for each K setting using the admixture model, with each simulation set to a 5000 burn-in period and 50,000 Markov chain Monte Carlo (MCMC) repetitions. To determine the optimal number of clusters [43], STRUCTURE HARVESTER [44], was used for calculate the Delta K statistical test in combination with the likelihoods (posterior probabilities) of each preset K. Results from simulations with the highest likelihood within each number of different K simulations were chosen to assign accessions to populations. Accessions with a population membership coefficient of less than 0.7 were identified as potential hybrids. 

Pairwise F_ST_ metrics [45] were also calculated in Genetix V4.05 [36] to estimate the divergence between the clusters identified by DAPC analysis. The value of F_ST_ vary from zero to one; when F_ST_ = 0 the subpopulations are identical, while when F_ST_ = 1 they are completely differentiated in relation to the fixation of different alleles in the subpopulations.

Differences of trait means were checked by a nested analysis of variance (ANOVA) based on the following sources of variation, between clusters, between germplasm types (landraces and elite varieties) within clusters, and between accessions among germplasm types within clusters. 

Principal component analysis (PCA) was performed to determine the overall morphological traits distinctiveness, and to investigate the relationships between the traits. Principal component analysis was performed using the software JMP version 8.0 [46]. 

## 3. Results

### 3.1. Overall Levels of Genetic Diversity

Forty-four SSR markers broadly distributed between the A and B durum wheat genomes were analyzed in the present study. All SSR markers showed polymorphism and a total of 242 alleles were detected across the 164 durum wheat genotypes. The average N_a_ per SSR was 5.50, ranging from two alleles (*Xgwm357*, *Xgwm165-4A*, *Xgwm415*, *Xgwm408*, and *Xgwm169*) to 14 alleles (*Xgpw2302*) (Supplementary Materials file 1: Table S3). Gene diversity or H_e_ across the total accessions was 0.607, and ranged from a low 0.105 (*Xgwm374*) to a high 0.832 (*Xgwm6*) (Supplementary Materials file 1: Table S3). 

When the whole germplasm collection was subdivided into two groups on the basis of the improvement status of each accession (accessions vs. varieties), a higher mean N_a_ per locus was observed in the accessions (N_a_ = 5.39) compared to the varieties (N_a_ = 3.86) (Table 1). FPTest [39] among accessions and varieties indicated significant differences in allelic counts (R_s_ = 4.562 and R_s_ = 3.830, *p* < 0.01). 

In order to evaluate how genetic diversity changed over time different groups of accessions were identified and compared. When considering the accessions, a slight, but not significant, increase of diversity was observed from Group1 (H_e_ = 0.579, R_s_ = 3.715) to Group2 (H_e_ = 0.603, R_s_ = 3.882), followed by a decrease in variation in Group3 (H_e_ = 0.531, R_s_ = 3.303). Nevertheless, when considering the varieties, a loss of genetic diversity was observed from the beginning of the 20th century to nowadays germplasm. Indeed, the highest H_e_ and R_s_ were observed in “old and intermediate” genotypes (H_e_ = 0.604, R_s_ = 3.434) as compared to “modern” varieties (H_e_ = 0.534, R_s_ = 3.011).

### 3.2. Genetic Structure of the Wheat Collection

The population structure of the germplasm collection, including both accessions and pedigreed varieties, was first assessed using a DAPC analysis, a method that identifies and describes clusters of genetically related individuals within a dataset [40]. The BIC analysis used to identify the optimal number of clusters clearly identified six genetic clusters (K = 6, Supplementary Materials file 1: Figure S1).

**Figure 1:**
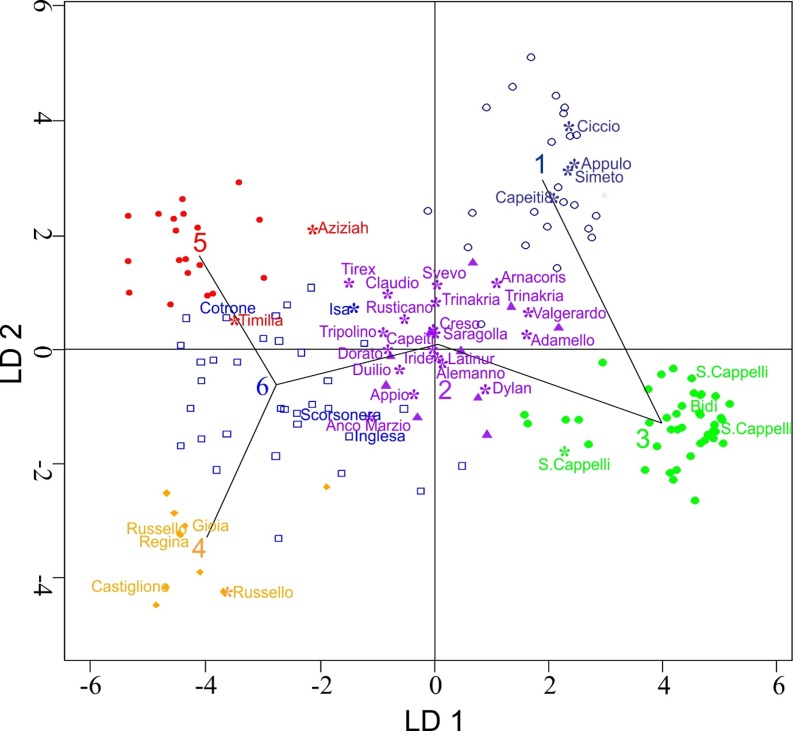
Scatterplot of the first two principal components of the DAPC applied on 136 ex situ accessions and 28 durum wheat varieties. Minimum spanning tree connects the six groups. Varieties (*) and named accessions are reported. Numbers and colors identify the clusters. LD: loadings.

To describe the relationships among the six clusters, a scatterplot of the first two principal components of the DAPC, which accounted for 28% of the total variance, is presented in Figure 1. The distribution of the ex situ accessions and the varieties among the six clusters is reported in Table 2. Germplasm ex situ accessions were not separated from varieties, but varieties representing a homogeneous genepool (related pedigrees) were included in the same cluster. Three clusters (C3, C4, and C6) included germplasm accessions and only one variety in each one; C5 and C1 included mostly germplasm accessions, with only two (C5) and four (C1) varieties. A large number of the analyzed varieties (*n* = 19) did cluster in C2 with 10 germplasm accessions (Table 2). The distribution of diversity over collection time shows how the pattern of substitution of old varieties by newly released varieties did occur. Additionally, accessions collected from (1947–1950) were never included in C1 and C2, accessions collected in (1973–1982) were present in each of the DAPC clusters and genotypes collected from 1983 to 2003 (third temporal group) were assigned to all but one cluster (C5) (Table 2). Clustering revealed that the choice of collecting sites was very careful at avoiding the most recently introduced varieties. Out of 136 accessions only 10 did cluster in C2 that included most “modern” varieties and 23 in C1.

The assignment of the varieties largely reflected the genetic basis and breeding strategies applied for their development. Cappelli, selected from the North African population “Jean Retifah” and considered one of the most relevant ancestors of the modern durum wheat gene pool, did cluster in C3. The same cluster also included two accessions (TRI3873 and TRI3882) collected in 1950 in Calabria that, according to the passport data information, were also named, “Senatore Cappelli” and the accession named “Bidi” that, according to De Cillis (1927), was from a variety from Tunisia, was not different from the one that originated Cappelli. Russello, a variety selected from a Sicilian local population, was the only variety included in C4 where six out of the ten accessions were collected in Sicily in 1950, including accession TRI3857 also named “Russello”, TRI3851 named “Gioia”, and TRI3855 named “Regina”, which are old Sicilian varieties. It should be noted that, according to molecular data, accessions and varieties with the same “variety” name that cluster in the same group do not have the same genotypes thus cannot be considered “duplicates”. Two old varieties, namely Aziziah, selected from the exotic landraces “Near East”, and Timilia, selected from Sicilian landraces, did cluster in C5 with accession TRI14111, named “Triminia”, collected in Campania in 1982. Capeiti 8, Appulo, Simeto, and Ciccio, that could be traced back to a common origin on the bases of pedigree information (Cappelli × Eiti 6), were assigned to C1 which also included accession TRI16687, named “Trinakria”, a variety that was obtained using Capeiti 8 as parent. Except for the variety Isa, that did cluster to C6 with accessions TRI3518 named “Cotrone”, TRI3519 named “Inglesa”, TRI3520 named “Scorsonera”, and TRI3853 named “Bianmaia”, which are also old varieties, all the remaining 19 varieties were assigned to C2. These genotypes were the most recently released varieties and were related, according to their pedigree information, to Creso, and to the innovative semidwarf materials developed at CYMMIT. Accession TRI13079 named “Creso” and TRI16565 named “Capeiti” were also included in C2.

**Figure 2:**
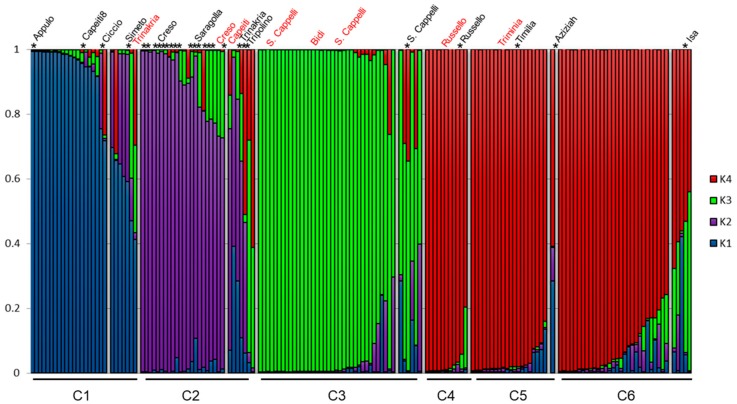
Population structure analysis for 136 accessions and 28 durum wheat varieties based on SSRs analysis. K-values of subpopulations are shown to right and accessions and varieties grouped according to the six clusters identified by DAPC are given below. Each individual is represented by a vertical line, and cluster assignments are indicated by color. Individuals are considered assigned to a cluster if their posterior probability in that cluster is at least 0.7. Varieties are indicated with a star.

To further describe the population structure of the whole germplasm collection, a Bayesian model-based clustering method implemented in the STRUCTURE program was performed. The number of subpopulations (K) was identified based on maximum likelihood and Delta K values [43]. The Delta K test suggested that our sample was made up of four main genetic groups (K = 4), with the next largest peak found at seven clusters (K = 7) (Supplementary Materials file 1 Figure S2). At K = 4, four clusters were clearly separated: the first cluster (K1; blue color in Figure 2) was composed of 17 accessions and three old-intermediate varieties; the second cluster (K2, pink color in Figure 2) included 16 varieties and only six accessions; the third cluster (K3; green color in Figure 2) included a total of 36 accessions and no varieties; and the fourth cluster (K4; red color in Figure 2) was composed of 58 accessions and only two old-intermediate.

The STRUCTURE bar graphic (Figure 2) also provides information on the level of admixture in the study sample. At K = 4, 138 genotypes out of 164 (84%) were assigned to one or another group with more than 70% posterior probability (Figure 2). The remaining 26 not assigned accessions (16%) were assumed to have a mixed ancestry.

**Figure 3:**
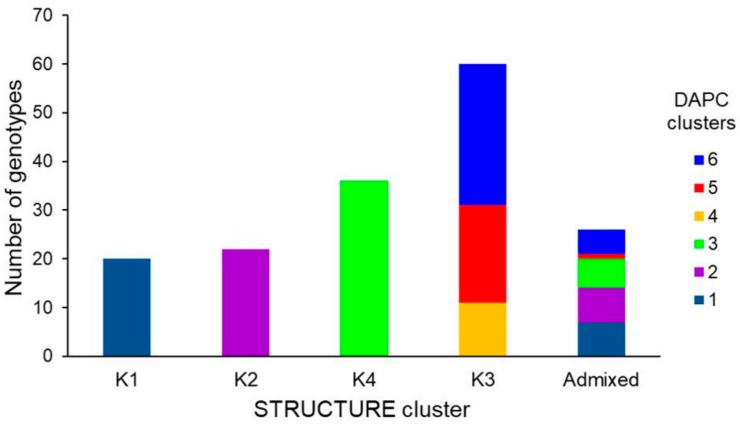
Comparison of individual assignment between DAPC and STRUCTURE analyses for 136 ex situ accessions and 28 durum wheat varieties based on 44 SSRs. Individuals are considered assigned to a cluster if their posterior probability in that cluster is at least 0.7.

Individual assignments provided by STRUCTURE differed only slightly from those by DAPC: one of the STRUCTURE clusters (K3) was split into three DAPC groups, while the other three clusters where consistent with the DAPC assignments (Figure 3).

### 3.3. Genetic Differentiation among DAPC Clusters

Genetic differentiation among the six DAPC clusters was investigated by computing pairwise F_ST_ values. All pairwise F_ST_ values were significant and ranged from 0.122 (C5–C6) and 0.502 (C3–C4), as shown in Table 3.

**Figure 4:**
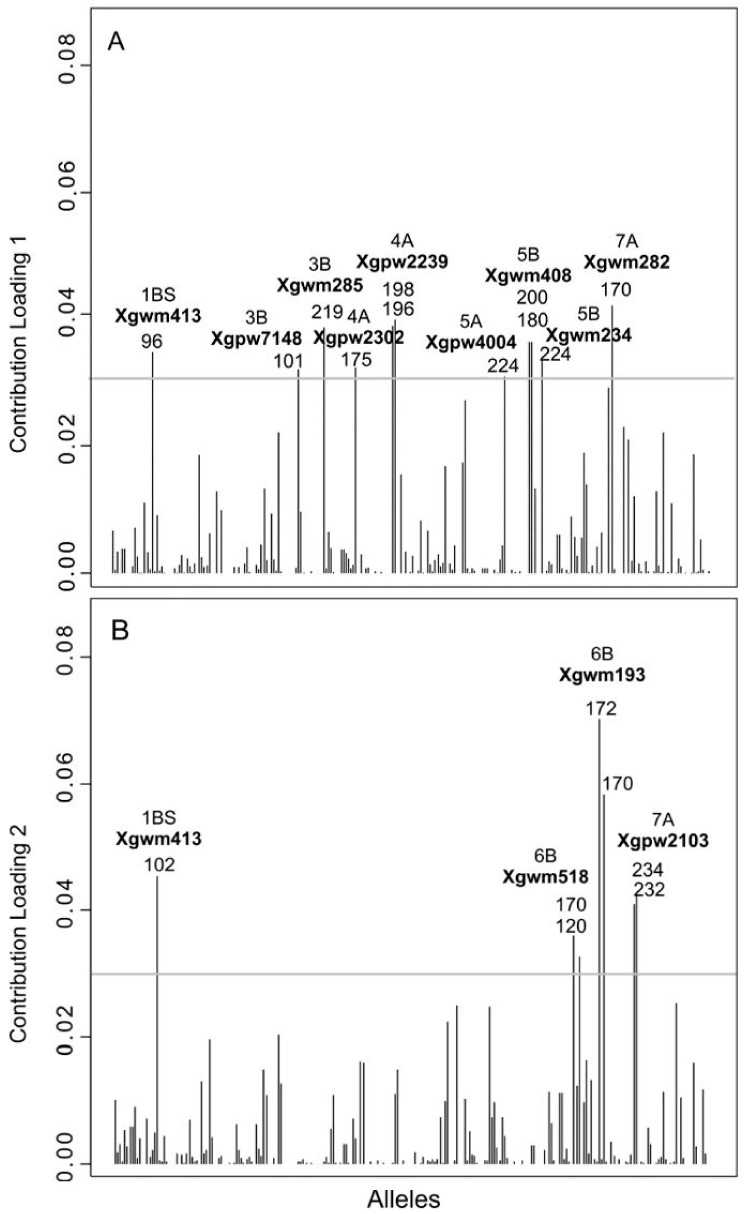
Contribution of alleles to the first (**a**) and the second (**b**) principal components of DAPC applied on 136 accessions and 28 durum wheat varieties. The height of each bar is proportional to the contribution of the corresponding allele to the first and second principal components of the analysis, respectively. Only alleles whose contribution was above a threshold (grey horizontal line) are indicated for the sake of clarity.

To get some insight into the underlying causes of the differentiation among clusters, we inspected the associated allele loadings. DAPC is perfectly adapted to this task, as it finds linear combination of alleles (the discriminant functions) which best separate the clusters. Alleles with the largest contribution to this discrimination are the most different among groups. Therefore, the plot of allele contribution (Figure 4) could be useful for a graphical assessment of alleles of major interest.

The locus *Xgwm408*, located on the long arm of chromosome 5B, mostly contributed to the first principal component with allele 180 and allele 200. The first allele discriminated C4 and C5, which was not represented, from the other clusters, whereas the second allele was not recorded in accessions attributed to C1 and C3. Allele 170 of locus *Xgwm282*, located on the long arm of chromosome 7A, was detected in all DAPC clusters except for C1 and C3. The locus *Xgwm234*, on the short arm of chromosome 5B, contributed to the first principal component with allele 224 detected only in accessions belonging to C1, C2, C3, and C6. The same discrimination pattern was observed for allele 175 of the locus *Xgpw2302*, located on the short arm of chromosome 4A.

An additional locus mapped on the short arm of chromosome 4A, *Xgpw2239*, contributed to the first principal component with two alleles, namely allele 196 and allele 198. The first allele was recorded in all clusters except for C4, while allele 198 was detected only in C3, C4, C5, and C6. The allele 219 identified at locus *Xgwm285* on the short arm of chromosome 3B was useful to discriminate C1, C2, and C3 from the remaining clusters identified by DAPC analysis, in which this allele was not recorded. Finally, allele 96 at locus *Xgwm413* (short arm of chromosome 1B), allele 101 identified at locus *Xgpw7148* (long arm of chromosome 3B), and allele 224 for locus *Xgpw4004* (long arm of chromosome 5A), showed similar behavior in discriminating DAPC clusters. Indeed, these alleles were all detected in C1, C2, C3, and C6.

When considering the second principal component, four loci showed the largest contribution to the discrimination among clusters. Regarding the locus *Xgwm193*, located on the short arm of chromosome 6B, two alleles showed a high contribution; allele 170 was recorded in all DAPC clusters except for C5, whereas allele 172 was identified in accessions assigned to all clusters except for C4. A similar level of discrimination was observed for an additional locus mapped on the short arm of chromosome 6B, namely *Xgwm518*. Allele 120 was observed in all clusters except for C4; allele 170 was useful to discriminate C5, whose accessions were not characterized by this allele, from the remaining clusters. The locus *Xgpw2103*, located on the short arm of chromosome 7A, contributed with allele 232 and allele 234. The first allele was not recorded in C3, whereas the second allele was not detected in C1, C4, and C5. Finally, allele 102 identified at locus *Xgwm413* (short arm of chromosome 1B), characterized genotypes attributed to C1, C2, C5, and C6.

### 3.4. Morphological Characterization

To identify the relationship between molecular SSR diversity and phenotypic characters of agronomic interest, a greenhouse phenotyping was conducted. Twelve morphophysiological characters were scored on three plants for each accession and variety.

The differences among the means of 12 characters were tested by a nested analysis of variance (ANOVA): between clusters, between germplasm type (accessions and varieties), and between accessions among germplasm type (Supplementary Materials file 1: Table S4). Differences among clusters were highly significant for all characters except for kernel width and kernels number/spike. For all remaining sources of variation means were always significantly different. For all the traits the summary statistics for each group (accessions and varieties) included in the analyzed germplasm are reported in Supplementary Materials file 1: Table S5. Ex situ accessions showed a higher mean heading date (38.87 days) than varieties (36.17 days) and were taller (mean plant height = 101.69 cm) than varieties (mean plant height = 72.09 cm). In accessions the spike was longer (19.53 cm) and had a higher number of spikelets (*n* = 21.73) than in varieties (18.06 cm and *n* = 17.40, respectively). Similarly, the number of fertile spikelets per spike was higher in accessions (*n* = 17.50) when compared to the varieties (*n* = 15.69). By contrast, kernel number per spikelet was lower in accessions (*n* = 1.68) than in varieties (*n* = 2.21). Additionally, higher values were recorded for both kernel weight/spike and 1000 kernels weight in accessions (1.95 g and 52.26 g, respectively) in comparison to the varieties (1.81 g and 47.42 g, respectively).

### 3.5. Morphological Differentiation among Discriminant Analysis of Principal Components Clusters

The six genetic structure clusters revealed by DAPC were further investigated to detect pattern of diversity for morphological traits of agronomic interest. A nested analysis of variance (ANOVA) was run. Means were significantly different (*p* < 0.001) between clusters for all the traits except for kernel width and kernels number/spike. Additionally, means were highly significant (*p* < 0.001) for all the traits between germplasm types within clusters and between accessions among germplasm types within cluster (Table 4).

The mean and the range of variation for the six clusters grouped according to DAPC are shown in Supplementary Materials file 1: Figure S3 along with the overall mean. The means and the ranges can be used to identify phenotypically divergent sources for traits of interest in breeding programs. For all traits within the six clusters groups different ranges were expressed. For some traits (e.g., heading date and fertile spikelets number per spike) the ranges expressed within cluster 2 (made of 19 varieties) were narrower than in other clusters, suggesting a low degree of diversity. Sampling from different clusters is expected to increase allelic diversity and selection response in breeding programs.

**Figure 5:**
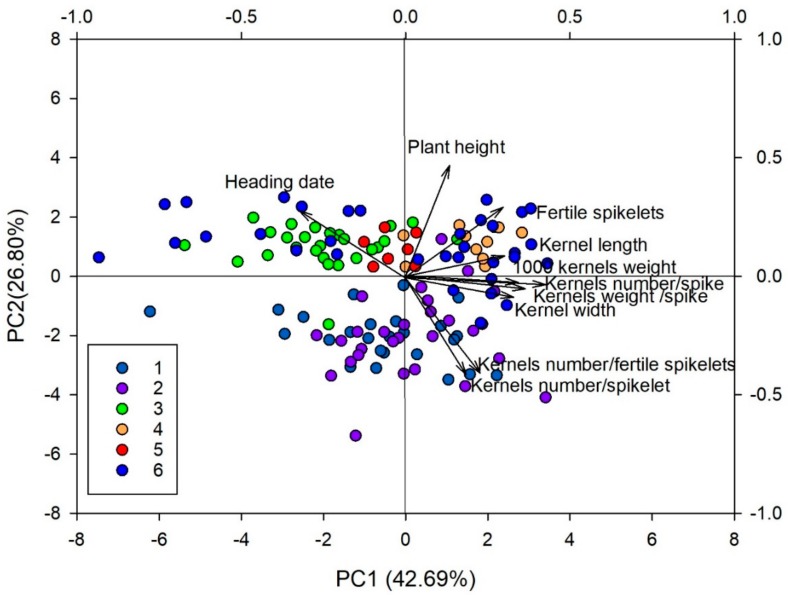
Principal component analysis (PCA) and cumulative proportion of total variance for the first and second PC for 136 accessions and 28 durum wheat varieties. Different colors are used for the six groups uncovered by the DAPC. Arrows indicate the loadings for each trait along the first two components.

Principal components analysis (PCA) analysis on all traits revealed four principal components (PC1–PC4) explaining 90% of total variance (Table 5). The first principal component (PC1) explained 42.7% of total variance and was mostly associated (higher loadings) with kernels weight/spike, kernels number/spike, 1000 kernels weight, kernel width, kernel length, and heading date, 26.8% of additional variation projected by PC2 came primarily from plant height and spikelets number/spike. PC3 and PC4 added, respectively, another 14.9% and 4.5% of the observed traits variability: these components accounted mainly for kernels number/spike, 1000 kernels weight, and kernel length and width (Table 5). A plot of genotype distribution for the first two principal components is presented in Figure 5.

Principal components analysis (PCA) analysis based on the measured morpho-agronomic traits was also able to differentiate contrasting wheat groups as identified using the DAPC analysis. Genotypes that did cluster in C1 and C2 with the DAPC analysis were also included in the same group in PCA analysis and, according to the first PCA loadings, were early heading and short plant height, while genotypes in C3, C4, C5, and C6 showed late heading, tall plant height, and low kernel number/fertile spikelet and kernels number/spikelet (Figure 5).

## 4. Discussion

The recent history of durum wheat has been characterized by the replacement of landraces at first with selected pure line varieties and after with modern cultivars obtained by crosses between lines that did also include lines from mutagenesis, exotic germplasm, and different wheat species. Luckily ex situ collections preserve precious genetic resources collected over time that include germplasm from different genepools. Since in most cases the passport data do not include genetic information, their effective use in agriculture and breeding programs is very low.

The identification of useful genotypes is largely improved if the genetic structure of the germplasm collection is known. In the present study, we combined molecular genotyping and plant phenotyping with the comparison to well-known pedigreed varieties to describe the genetic structure of durum wheat germplasm collected in Southern Italy and preserved ex situ.

### 4.1. Diversity of South Italian Durum Wheat Germplasm

Overall, gene diversity for SSR markers was 0.607, and within the range of previous studies in durum wheat [9,10,11,47]. Gene diversity and allelic richness within ex situ accessions were higher than within pedigreed varieties. This indicates a substantial loss of genetic diversity over time [48] that might involve alleles valuable for plant improvement and future demands of producers and consumers. Moreover, the gene diversity within accessions and within varieties is representative of different genepools. In fact, most modern varieties, that are the results of hybridization and also include newly introduced germplasm cluster in group C2 (*n* = 16) or in C1 (*n* = 2), while most accessions cluster in four different groups (C3, C4, C5, and C6) that include “landraces” or varieties from pure line selection largely representative of the Italian–Mediterranean genepool. In ex situ accessions a small increase of diversity was observed from Group 1 (*n* = 37 accessions collected from 1947 to 1950) to Group 2 (*n* = 77 accessions collected from 1973 to 1982). Group 2 accessions, which were included in all clusters, were “old” and “new” germplasm grown at the same time in the same area at the time when intense varietal substitution was in progress. As expected, the amount of collected diversity decreased in the following time (*n* = 22 accessions collected from 1983 to 2003) when most obsolete varieties had been already replaced. Clustering also showed that the choice of collecting sites was very careful to avoid the most recently introduced varieties. Out of 136 accessions, only 10 did cluster in C2 that included most “modern” varieties, and 23 in C1 which includes varieties derived from hybridization.

It has been argued that the selection pressure applied in breeding programs may have reduced the level of genetic diversity in durum wheat germplasm [49]; in our study modern varieties (1974–2007) did show a degree of genetic diversity lower than in the “old and intermediate” group (1915–1973).

Our findings on the elite germplasm agree with previous reports by Medini et al. [50], Reif et al. [48], and Figliuolo et al. [11], however, Maccaferri et al. [10] did show a progressive increase of the genetic basis in the elite durum wheat germplasm. Moreover, Martos et al. [19] and Laidò et al. [13] observed that the overall molecular diversity of durum wheat remained quite constant throughout genetic improvement occurring during the 20th century. The reduction of diversity might be explained by the “Green Revolution”, which was characterized by breeding semidwarf varieties with a higher yielding potential due to an increased harvest index and better lodging tolerance, particularly under high fertilizer and water inputs [6]. These new high-yielding semidwarf modern varieties were based on a limited number of founder genotypes and rapidly dominated the wheat germplasm base [48,51]. This assumption may suggest that a high proportion of SSRs markers used in this study may be associated with chromosomal regions selected during breeding programs. These could be chromosomal regions harboring some agronomic relevant trait loci (such as those determining semidwarf habit or yield) that resulted in more uniform and stable modern varieties.

The now available high-density consensus maps of durum wheat coupled with sequencing information provide an advanced tool [52,53] for durum wheat fingerprinting. Indeed, the increasing knowledge of polymorphic marker distribution in the durum wheat genome will facilitate the selection of SSR makers useful to detect pattern of diversity associated to different gene pools and will improve the use of ex situ collections in breeding programs designed to exploit opportunities offered by the wider wheat gene pool.

### 4.2. Analysis of Population Structure

Discriminant Analysis of Principal Components and STRUCTURE analysis were very informative and complementary about the structure of the durum wheat ex situ germplasm collection and were useful to identify the most diverse accessions for utilization in low input agriculture and in breeding programs. For instance, accessions were included in all clusters, whereas modern varieties from hybridization, including in their pedigree the innovative semidwarf CIMMYT materials, were assigned to only two clusters (C1 and C2). These findings are consistent with the repeated use of a few founder genotypes that played a relevant role in the creation of the genetic basis of modern genetic pools, in turn becoming progenitors of new elite varieties and completely replacing traditional varieties or landraces [5,54]. The continued use of these genotypes made the gene pool smaller for all of the durum wheat elite varieties and resulted in the loss of genetic diversity [13]. Low levels of genetic diversity of Italian cultivars developed by Italian breeders were also observed by Kabbaj et al. [18]. The low lewel of genetic diversity can be explain as the combined result of frequent hybridization of a reduced number of founders and the strong selection pressure for the same trait needed for Italian growing conditions and the requiraments of the pasta industry [18].

New variability is needed to face the challenges of modern low input agriculture. Accessions from the ex situ collection that are genetically distant, as those included in different clusters, could be useful for this purpose. Pedigreed varieties were useful to identify the gene pool of origin of the accessions that did cluster in different groups. It should be noted that the varietal purity was higher for those released more recently, while genotypes associated with older releases might be different from accessions with the same name. In some cases, Saragolla, a brand new variety that has been released in 2004 with an old name, or variety and accessions with the same name as “Russello”, might be selected as pure lines from the same “landrace” (Russello 329-S.97-S.G.7) [21], or the variety Senatore Cappelli, that now is very popular in organic agriculture, is genetically distinct from the two ex situ accessions with the same name, that cluster in the same group. The accession variety name must be also carefully verified: the Capeiti 8 variety is included in C1 as expected according to the pedigree, while the accession named “Capeiti”, which was probably misnamed, did cluster in C2 and is genetically admixed. Also, the Trinakria variety (C2) and “Trinakria” accession (C1) were included in different clusters, but in this case, the mixed genotype was the variety. The Tripolino that we analyzed was probably not the “old” variety since it did cluster in C2 with most modern varieties while the synonymous variety Azizia did cluster in C5. Moreover, the STRUCTURE analysis revealed that it is actually an admixed genotype, thus suggesting that seeds of “pedigreed” varieties of “true” sources must be carefully tested for varietal purity. Two accessions and one variety with name Senatore Cappelli cluster in C3. The two accessions look fairly similar genetically and are also similar to Bidi that originated from the same “landrace”, while the now grown Senatore Cappelli variety is genetically distinct and shows some degree of admixture with the old germplasm from Sicily and with C1 and C2 where the most advanced varieties are included. It seems that evolution through natural hybridization took place for Senatore Cappelli and that the pure line, recently reselected for distribution to farmers, is actually different from the old one. These results were also confirmed by phenotypic data that show Senatore Cappelli variety is different from both accessions and from Bidi: the plant is shorter and has later heading, longer head, less fertile spikelets, smaller kernels, and fewer kernels per spike.

In a recent study [18], a panel of 370 durum wheat genotypes, including 35 Italian genotypes (29 elite variety and 6 landraces), were genotyped using SNP markers. The Italian genotypes were attributed to seven subclusters. Three “landraces” did cluster with elite varieties indicating that these accessions were probably not “landraces” but cultivars that were not properly identified during the collecting missions [18]. For the other three Italian “landraces” no information about their origin was available in passport data so a direct comparison with the accessions used in this study was not possible. Out of twenty-nine Italian elite varieties studied by Kabbai et al. [18] only five were common to our study (Capeiti 8, Creso, Applo, Claudio, and Svevo). Capeiti 8, an “old” Italian variety from a cross between Syriacum × Mediterraneum groups (Eiti 6 × Cappelli), did cluster with lines and cultivars from the International Center for Agricultural Research in the Dry Areas (ICARDA) breeding program that include the old cultivar “Om Rabi” in their pedigree [18]. Also in our study was Capeiti 8, which did cluster with “old” genotypes selected from indigenous and exotic landraces. The remaining four varieties were included in three different clusters in Kabbai et al. [18], while in our study they did cluster in the same group with the “modern” dwarf genotypes released after 1974 that were selected from crosses that also included CYMMIT breeding lines.

Discriminant analysis of principal components can be also used to identify the alleles with the largest contributions to the discriminant functions, as an approach to detect putative patterns among the genes determining the group differentiation [40]. A plot of SSR allele contributions was used to identify alleles of major interest and, substantially, most of them were detected at loci associated to quantitative trait locus (QTLs) for phenological traits, as well as to resistance to wheat diseases and to grain qualitative parameters. Indeed, when analyzing the alleles’ contributions to the first principal component, the gene *Vrn-B1*, affecting the vernalization response, was found to be tightly linked to the marker *Xgwm408* [55,56]. Noteworthy, the genes of the *Vrn* system are the most important to determine the rate of generative development in wheat and, thereby, its flowering time [55,56,57]. Additionally, a QTL for Fusarium head blight (FHB) resistance was mapped proximal to marker *Xgwm282* [58], whereas the locus *Xgwm234* was found to be significantly associated with dough strength [59,60] and the marker *Xgwm285* was located at only 2.1 cM from the *tsn2* gene, conditioning resistance to race 3 of the fungus *Pyrenophora tritici-repentis* (Died.), causal agent of tan spot in durum wheat and inducing necrosis [61]. Since the inspection of alleles for the abovementioned markers showed that C1 and C3 were highly differentiated from C4 and C5, this could be an effective criterion for selection.

Similarly, when considering the alleles’ contribution to the second principal component, we noticed that the marker *Xgwm193* encompasses the peak of the *QGpc.ndsu.6Bb* QTL for grain protein content. Thus, it could be useful to use this SSRs marker to select for the high-GPC allele in Marker-Assisted Selection programs [62,63]. Finally, the marker *Xgwm518* was found to be linked to the *QLr.caas-6BS.2* QTL for resistance to leaf rust caused by *Puccinia triticina* Westend [64] and the marker *Xgwm413* to be associated to a QTL for grain weight on the short arm of chromosome 1B [65]. These three markers differentiated C3 and C4 from C5, allowing to highlight the potential of the DAPC method to go beyond mere group delimitation and to identify accessions potentially useful in breeding programs.

Genetic groups identified by DAPC were also differentiated on the basis of morphological traits, as confirmed by PCA. Moreover, accessions and varieties assigned to the same cluster, although genetically related on the base of molecular markers, exhibited different morphological traits. These results could be helpful to select, especially from C3 and C4, early-heading landraces well adapted to the Southern Italian semi-arid conditions, where earliness allows farmers to better tackle the drought season [16]. Within each of the six DAPC clusters accessions plants were taller than varieties. Plant height could be a preventive measure to face weed competition, which remains one of the main problems in organic wheat crops since no herbicides are allowed [66]. Indeed, durum wheat landraces, characterized by tall plants and early vigor, could provide early groundcover which is vital to compete for light, water or nutrients. This trait represents a competitive advantage over early emerging weeds and their suppression [67]. Finally, higher values for yield components in ex situ accessions in C3, C4, and C5 could be due to higher N-use efficiency and their ability to produce high yields at low soil-N availability typical in organic and low-input agricultural systems [68,69,70]. Therefore, since wheat landraces have evolved mostly in environments with low nutrient availability [71], they represent a source of variation for selection of varieties adapted to cropping systems with low fertilizer input.

## 5. Conclusions

Both the morphological traits and SSR markers used in the present study were equally appropriate to provide a first overview of the genetic diversity levels and of the population structure within the whole durum wheat germplasm collection from Southern Italy.

Evolution through natural hybridization is suggested for old varieties recently reintroduced in agriculture and subjected to careful conservative selection by pure line.

Despite similar level of diversity for molecular markers in ex situ accessions and varieties DAPC did show that they cluster in different groups, thus representing different genepools capable to provide different sources of genes.

The analysis of diversity based only on molecular markers must be supported by passport data and or some phenotyping or knowledge in crops. In crops where plant breeding played a relevant role, social factors that promote seed exchange might be as significant as ecological factors in determining the distribution of genetic diversity and variation in such “ex situ” collections might be heavily structured.

The germplasm evaluation highlighted the existence of a broad genetic base in ex situ accessions and a narrowing of diversity in varieties due to breeding activities. The investigation of population structure suggested the genetic potential of landraces for the detection of unexplored sources of variation and allowed to identify groups of accessions differentiated both at molecular level and for morphological, phenological, and qualitative traits not directly evaluated and potentially useful in sustainable production systems.

The generated knowledge about the levels of diversity and population structure could be an important contribution for parent selection in durum wheat breeding programs and for germplasm management and conservation. 

## Figures and Tables

**Table 1:** Genetic diversity estimates for 136 ex situ accessions per collection year and 28 durum wheat varieties per year of release.

Germplasm Group	*n*	N_a_	H_e_	R_s_
Accessions	136	5.39	0.601	4.562
Group1 (1947–1950)	37	4.34	0.579	3.715
Group2 (1973–1982)	77	5.00	0.603	3.882
Group3 (1983–2003)	22	3.57	0.531c	3.303
Varieties	28	3.86	0.576	3.830
Old and intermediate varieties (1915–1973)	9	3.45	0.604	3.434
Modern varieties (1974–2007)	19	3.09	0.534	3.011

*n* = number of samples; N_a_ = mean number of alleles per locus; H_e_ = expected heterozygosity; R_s_ = allelic richness computed for a minimum sample size of nine diploid individuals per group.

**Table 2:** Number of accessions per collection year and varieties per year of release in six clusters obtained by discriminant analysis of principal components (DAPC) for 136 ex situ accessions and 28 durum wheat varieties.

Germplasm	Year	C1	C2	C3	C4	C5	C6	Total
Accession	Collection							
Group1 (1947–1950)	1947–1950			16	7	2	12	37
Group2 (1973–1982)	1973–1982	17	5	19	1	17	18	77
Group3 (1983–2003)	1983–2007	6	5	6	2	-	3	22
Total		23	10	41	10	19	33	136
Varieties	Release							
Old and intermediate	1915–1973	2	3	1	1	2	-	9
Modern	1974–2007	2	16	-	-	-	1	19
Total		4	19	1	1	2	1	28

**Table 3:** Differentiation indices (F_ST_) between all pairwise combination of clusters identified by DAPC performed on 136 durum wheat ex situ accessions and 28 varieties.

DAPC Cluster	C1	C2	C3	C4	C5	C6
C1		0.225 **	0.284 **	0.415 **	0.317 **	0.220 **
C2			0.256 **	0.276 **	0.218 **	0.145 **
C3				0.502 **	0.458 **	0.327 **
C4					0.251 **	0.158 **
C5						0.122 **
C6						

(** significant for *p* < 0.01).

**Table 4:** Analysis of variance (ANOVA) of 136 durum wheat ex situ accessions and 28 varieties for 12 morphophysiological plant characters comparing the six clusters identified by DAPC, the germplasm types (accessions and varieties) within clusters, and the accessions within type group and cluster.

	Source of Variations			
Traits	Between Clusters	Between Germplasm Types (Clusters)	Between Accessions (Germplasm Type × Clusters)	Error
Heading date (from 10 April)	268.1 ***	186.2 ***	126.4 ***	8.7
Plant height (cm)	13,786.8 ***	394.1 ***	519.5 ***	95.1
Spike length (cm)	98.7 ***	28.9 ***	9.4 ***	2.2
Spikelets number/spike (*n*)	244.2 ***	47.6 ***	16.9 ***	1.8
Fertile spikelets number/spike (*n*)	81.9 ***	25.2 ***	20.3 ***	6.3
Kernel length (mm)	1.7 ***	0.6 ***	0.7 ***	0.1
Kernel width (mm)	0.06 ns	0.3 ***	0.4 ***	0.05
Kernels number/spike (*n*)	263.0ns	39.1 **	182.4 ***	65.4
Kernels weight/spike (g)	1.7 ***	1.1 ***	1.0 ***	0.2
1000 kernels weight (g)	370.7 ***	569.9 ***	323.9 ***	46.5
Kernels number/spikelet (*n*)	2.6 ***	0.7 ***	0.3 ***	0.1
Kernels number/fertile spikelet (*n*)	1.2 ***	0.5 ***	0.2 ***	0.1

(**, *** significant for *p* < 0.01; *p* < 0.001. ns = not significant).

**Table 5:** Correlations between four principal components variables and 12 original morphophysiological plant characters for six groups obtained from 136 durum wheat “ex situ” accession and 28 varieties.

Principal Components	PC1	PC2	PC3	PC4
Kernels weight/spike	**0.42**	−0.04	−0.01	0.11
Heading date	**−0.31**	0.27	0.21	0.37
Plant height	0.14	**0.46**	0.08	−0.14
Spikelets number/spike	0.23	**0.43**	−0.03	−0.02
Kernels number/fertile spikelets	0.22	**−0.42**	0.25	0.04
Kernels number/spikelet	0.19	**−0.41**	0.38	0.09
Kernels number/spike	**0.37**	−0.05	**0.39**	0.07
Spike length	0.17	0.29	**0.34**	−0.25
Fertile spikelets number/spike	0.29	0.29	**0.29**	0.03
1000 kernels weight	**0.33**	−0.03	**−0.44**	0.06
Kernel length	**0.30**	0.09	−0.28	**0.72**
Kernel width	**0.34**	−0.08	−0.35	**−0.48**
Eigenvalues				
Variation explained (%)	42.7	26.8	14.9	4.5
Cumulative proportion of total variance	42.7	69.5	84.4	88.9

In bold, variable loading scores with the greatest loads on each component.

## Supplementary Materials

The following are available online at http://www.mdpi.com/2073-4425/9/10/465/s1, Figure S1: Bayesian Information Content (BIC) for increasing values of the number of clusters. The chosen number of clusters was K = 6, Figure S2: STRUCTURE estimation of the number of subpopulations for K ranging from 2 to 10 by mean likelihoods (A) and Delta K values (ΔK) (B), Figure S3: Means and ranges of variation for 12 morphophysiological plant characters for six DAPC group clusters obtained from 136 durum wheat accession (in black) and 28 varieties (in red), Table S1: List of durum wheat accessions used in the study. Seed source, collecting date, geographical origin, and DAPC cluster attribution of each accession are reported, Table S2: List of durum wheat varieties used in the study. Year of release, pedigree, breeder, and seed source of each variety are reported, Table S3: Primers sequence and characteristics of the 44 SSR markers used to study diversity in a durum wheat germplasm collection from Southern Italy, Table S4: ANOVA analysis for the morphological traits measured on the wheat germplasm collection. Variation: between germplasm type (accessions and varieties) and between accessions among germplasm type (*, **, *** significant for *p* < 0.05; *p* < 0.01; *p* < 0.001, Table S5: Summary statistics with mean values, coefficient of variation (CV), minimum (Min), and maximum (Max) for morpho-quantitative traits measured on 136 accessions and 28 varieties.
